# Exploring a targeted epigenetic clock based on mortality-associated CpGs as a potential biomarker for frailty

**DOI:** 10.1186/s13148-026-02196-9

**Published:** 2026-07-07

**Authors:** Jonathan Awuah, Dhayana Dallmeier, Felix Boehm, Laura Herdtle, Dietrich Rothenbacher, Juan-Felipe Perez-Correa, Wolfgang Wagner

**Affiliations:** 1 Institute for Stem Cell Biology, RWTH Aachen University Medical School, Aachen, Germany; 2 Helmholtz Institute for Biomedical Engineering, RWTH Aachen University Medical School, Aachen, Germany; 3 Institute for Geriatric Research, Ulm University Medical Center, Ulm, Germany; 4 Department of Research on Ageing, AGAPLESION Bethesda Clinic Ulm, Ulm, Germany; 5 Department of Epidemiology, Boston University School of Public Health, Boston, USA; 6 Institute of Epidemiology and Medical Biometry, Ulm University, Ulm, Germany; 7 Center for Integrated Oncology Aachen Bonn Cologne Düsseldorf (CIO ABCD), Aachen, Germany

**Keywords:** Biomarkers, Biological age, Epigenetic clocks, DNA methylation, Frailty, Second generation clocks, Digital PCR

## Abstract

**Background:**

Age-related DNA methylation changes are promising biomarkers to track the individual aging process. Particularly second-generation epigenetic clocks capture aspects of biological age more accurately, but the requirement of genome wide profiles hampers implementation into practice. We therefore aimed to develop a simplified, targeted approach based on individual age- and mortality-associated CpG sites measurable by digital PCR.

**Results:**

We selected three CpG sites strongly associated with all-cause mortality in the Lothian Birth Cohorts and with chronological age in multiple publicly available repositories to establish the targeted age- and mortality-associated epigenetic clock (TaM clock). For comparison, we applied a previously published three-CpG signature selected solely for correlation with chronological age (TaC clock). These signatures were initially benchmarked using DNA methylation profiles from 20 frail and 20 non-frail participants of the ActiFE cohort. In fact, in this subset the TaM clock revealed significant association between the delta age and the frailty status based on a 32-item frailty index. Furthermore, the TaM clock outperformed the TaC clock at capturing a significant increase in epigenetic age in Down syndrome, Werner syndrome and HIV. We subsequently developed digital PCR assays to analyse 446 samples from the ActiFE cohort. One TaM clock site (cg20595453) showed significant association with both mortality and frailty. However, predictions generated by either targeted clock were not significantly associated with mortality and there was no clear relationship with frailty or age-related clinical parameters in this larger cohort.

**Conclusion:**

Our targeted signatures were not sensitive enough to reliably predict frailty or mortality in a relatively healthy study population, which seems to be a general challenge for epigenetic clocks. It may be necessary to include additional disease-associated target sites to support frailty analysis in personalized medicine.

**Supplementary Information:**

The online version contains supplementary material available at 10.1186/s13148-026-02196-9.

## Background

The human aging process shows pronounced interindividual differences and the concept of frailty has been suggested as an operational approach to take this variation into consideration [1]. Frailty is defined as a syndrome characterized through a physiological decline and an increased vulnerability to adverse health outcomes, and can be quantified using frailty indices [2, 3]. As such, a 32-item frailty index (32-item FI) was validated as a robust predictor of mortality in the ActiFE cohort—a population-based study of older adults from the greater Ulm area [4]. Nevertheless, comprehensive assessment via such indicators is often impractical in clinical settings [5]. This underscores the need to develop robust and simple biomarkers that can serve as simple proxies for frailty.

Generally, epigenetic clocks are biomarkers that are based on DNA-methylation (DNAm) changes at genomic cytosine-phosphate-guanine (CpG) sites—and they not only reflect chronological age but may also capture aspects related to mortality risk and morbidity [6]. First-generation clocks are primarily constructed to predict chronological age with high accuracy, whereas second-generation clocks such as PhenoAge [7] and GrimAge [8] incorporate CpG sites linked to health-related clinical variables, such as blood-cell composition, or plasma protein levels with the aim to predict phenotypic age or mortality risk, respectively. Thus, second-generation clocks have been shown to track responses to therapeutic interventions better [9]. Furthermore, Li et al. introduced the epigenetic Frailty Risk Score (eFRS), a biomarker derived directly from frailty-associated CpG sites, which showed moderate correlation with frailty throughout their follow-up [10]. Overall, these epigenetic signatures appear as promising candidates for measuring biological age or frailty, however, definitive conclusions regarding their utility in precision medicine remain elusive, which is in part due to technical and scientific challenges regarding generalizability, reliability, and interpretation [11]. Another hurdle for most second-generation clocks is their reliance on DNAm profiles encompassing hundreds of CpG sites, which typically necessitates genome-wide analysis platforms, such as the Illumina Infinium BeadChip arrays. These clocks can therefore hardly be adapted to clinical settings, which requires methods that are fast, cost-effective, and approved for clinical diagnosis [12].

To overcome this limitation, several researchers have developed targeted clocks that rely on very few specific CpG sites and can still predict chronological age with high accuracy [13]. Because the number of required sites is lower, often below 10 CpGs, these targeted assays can be implemented with inexpensive, high-precision techniques such as pyrosequencing or digital PCR (dPCR) [14]. Such targeted signatures are a trade-off: on the one hand epigenetic clocks based on hundreds of CpGs may be more robust, but the methods for targeted measurement may reach higher accuracy of DNAm levels at individual CpGs [15], have less batch-to-batch variation, and do not require bioinformatic preprocessing [12]. Consequently, targeted epigenetic biomarkers would be easier to translate into a clinical setting, but their association with frailty parameters may be lower. To our knowledge, a targeted clock that is specifically trained on mortality-associated CpGs has not been described yet.

In earlier work we identified a subset of CpG sites in Illumina BeadChip data of the Lothian Birth Cohorts of 1921 and 1936 (LBC1921 and LBC1936) that are simultaneously linked to all-cause mortality and display age-related hypomethylation, suggesting that they may serve as biomarkers of aging [16]. Building on these findings, we constructed a targeted epigenetic clock that incorporates three of these age- and mortality-associated CpGs (TaM clock). As a benchmark, we employed a previously established targeted clock that utilizes three CpGs that were primarily selected for their association with chronological age (TaC clock) [14, 17]. Both targeted clocks were tested in datasets of frail and non-frail donors and of diseases associated with accelerated biological aging. Furthermore, we developed dPCR assays for these clocks and analysed 446 samples from the ActiFE cohort to evaluate the performance of the TaM and TaC clocks in comparison with clinical parameters and mortality.

## Methods

### Derivation of targeted epigenetic clocks

In our previous work, we identified 25 CpG sites that showed a high correlation with chronological age across various public DNAm datasets and that were significantly associated with all-cause mortality in both of the Lothian Birth Cohorts of 1921 and 1936 (LBC 1921 and LBC 1936, respectively) [16]. These 25 sites were selected without correction for leucocyte composition or preference for sites with homogenous methylation across cell populations. To further narrow down the selection, we focused on the three CpGs that had the highest correlation (Pearson’s r^2^) with chronological age in the public dataset GSE246337 (*n* = 500, ages 18–88, 51.8% women) [18]. The data was retrieved from Genome Express Omnibus (GEO) via the *getGEO* function from the R package *GEOquery* [19]. Following this procedure, we focussed on the CpG sites cg19453093, cg13823169, and cg20595453 for the TaM clock. As a benchmark for this type of signature, we used a targeted epigenetic clock based on three CpGs that were selected mainly for their correlation with chronological age and relatively low heterogeneity of DNAm differences across leucocyte subsets [14, 17]. This targeted chronological (TaC) clock utilizes the CpGs cg19283806, cg22454769, and cg17861230. To directly compare these clocks in selected samples from our testing cohort, they were trained on the independent dataset mentioned above (GSE246337) to generate multivariable linear regression models using chronological age as the outcome variable:

$$ \begin{aligned} {\text{TaM clock }}\left( {{\mathrm{BeadChip}}} \right) & \approx 212.11- 87.85 \times {\mathrm{cg}}19453093\\ & \quad - 117.76 \times {\mathrm{cg}}13823169\\ & \quad - 93.73 \times {\mathrm{cg}}20595453 \\ \end{aligned} $$

$$ \begin{aligned} {\text{TaC clock }}\left( {{\mathrm{BeadChip}}} \right) & \approx 35.02 - 73.06 \times {\mathrm{cg}}19283806 \\ & \quad+ 70.73 \times {\mathrm{cg}}22454769 \\ & \quad+ 54.02 \times {\mathrm{cg}}17861230 \\ \end{aligned} $$

These clocks were subsequently tested on a separate public dataset of Illumina BeadChip data (GSE40279) [20]. The individual sites of both the TaM and TaC clock were further examined for their methylation patterns and age associations across different leucocyte populations using the public methylation dataset GSE252045 [21].

### ActiFE cohort

For this study, we utilized samples of participants of the Activity and Function in the Elderly study (ActiFE study) [22, 23], a population-based cohort (*n* = 1506) of non-institutionalized older adults from the wider Ulm area in Germany. Included were all participants with available DNA samples isolated from whole blood at the time of baseline evaluation and frailty data at second follow-up after seven years (*n* = 446, ages: 65–88 years, 43.3% women). Their frailty at baseline was estimated via 32-item Frailty Index (32-item FI; 55 (12.3%) frail, 323 (72.4%) non-frail, and 68 (15.2%) missing data on the FI) [4] and the previously recommended threshold of 0.2 was used to delineate frailty (individuals with FI > 0.2 were considered frail) [24]. All study participants gave their informed written consent and approval for the study was given by the ethical committee of Ulm University (application no. 318/08 and 50/12).

### DNA methylation profiling of selected frail and non-frail samples

To benchmark the TaM and TaC clocks in comparison with other larger epigenetic clocks, we generated DNA methylation profiles with extreme frail or non-frail predictions using Illumina BeadChip technology. To select these samples, the cohort was divided by sex and ranked by 32-item FI. We selected the 10 individuals with the highest and lowest FI for each sex respectively, resulting in a subcohort of 40 individuals. The genomic DNA was bisulfite-converted and hybridized to Infinium MethylationEPIC v2.0 BeadChip arrays (Illumina, San Diego) at Life and Brain (Bonn, Germany). Quality control, preprocessing and normalization of the raw beta values was performed with the *ENmix* package in R (ENmix background correction [25], RELIC dye-bias normalization [26], and Regression on Correlated Probes (RCP) probe-type bias adjustment [27]). Probes with detection *p*-values > 0.01 or more than 10% of missing values were filtered out. Additionally, CpGs in sex chromosomes and single-nucleotide polymorphism (SNP) probes were excluded. Approximate leukocyte compositions were estimated for frail and non-frail participants using the ENmix implementation of the Houseman blood cell proportion estimation method [25, 28] and subsequently compared between groups.

The TaM and TaC BeadChip clocks were applied as described above. For comparison, we used the *methylCIPHER* R package [29–31] to calculate the epigenetic age with the Horvath [32], Zhang 2019 [33], PhenoAge [7], GrimAge version 2 [8], PC-PhenoAge, and PC-GrimAge [34] clocks. Age predictions using the Weighted 2D-Kernel Density Estimator (WDKE) and its variation score were calculated with the R scripts provided in the original publication [16]. Furthermore, we calculated the epigenetic Frailty Risk Score (eFRS) as previously described by Li and coworkers [10], but we could not include cg03725309 as it is not featured on the MethylationEPIC v2.0 array. For all of these signatures, no adjustment for blood cell composition was performed. Via the *t.test* function in R, two-tailed Welch’s t-tests were used to investigate differences in delta ages (predicted age—chronological age) and eFRS between the frail *versus* non-frail group. Outliers (predictions falling outside the interquartile range (IQR) ± 1.5 × IQR) for the frail or non-frail group respectively were excluded from the analysis for all clocks, since we anticipated that there might have been sampling errors. Respectively, we excluded one outlier for the TaM, PhenoAge, and PC-PhenoAge clocks, two for the TaC clock and WDKE, and four in the PC-GrimAge clock, all part of the non-frail subgroup. The correlation estimates (Pearson’s r) and confidence intervals of the 32-item frailty index with DNAm at the TaM and TaC sites, epigenetic clock predictions, and the eFRS were determined via the *cor.test* function in R.

### DNA methylation datasets of diseases with accelerated aging

To further explore how the selected CpGs are affected in diseases with signs of accelerated aging, we used datasets of Down syndrome (GSE52588) [35], Werner Syndrome (GSE131752) [36], progeroid laminopathies (GSE182991) [37], and of infections with human immunodeficiency virus (HIV; GSE67751) [38]. All of these datasets comprised control and diseased samples. Already processed beta value matrices were downloaded directly from their GEO repositories for the included studies, using the *getGEO* function [19]. To correct for methylation changes due to chronological age, we used the public dataset GSE246337 (500 blood samples of healthy donors) [18] to calculate linear regression models. For the individual CpGs of the TaM and TaC clocks, we then determined the deviation of these age-related DNAm levels (ΔDNAm), whereas for the BeadChip TaM and TaC clocks we determined the deviation of age-predictions (delta ages; ΔAge). Differences in disease compared to control samples were evaluated via two-tailed t-test.

### Methylation measurement via duplex digital PCR

Genomic DNA of peripheral blood samples was measured via NanoDrop2000 spectrophotometer (Thermo Scientific, Wilmington, United States), and approximately 500 ng of gDNA were bisulfite-converted using the Zymo Research Group EZ DNA Methylation Kit (Zymo Research, Irvine, United States). For the three TaM clock CpGs (cg19453093, cg13823169, cg20595453) we designed PCR primers and fluorescent-dye TaqMan probes that specifically discriminate methylated from unmethylated DNA using Zymo Research’s bisulfite-primer design tool (Irvine, USA). For two of the CpGs of the TaC clock, the dPCR assays were already established [14], and thus, we only had to design primers for cg22454769 (FHL2; Supplemental Table 1). All utilized primers and probes were ordered from Metabion (Planegg, Germany) and methylation measurements were obtained via duplex digital PCR assays on the QIAcuity One Digital PCR System (QIAGEN, Venlo, Netherlands). For all assays, a three-step PCR protocol of denaturation (95 °C), annealing (55 °C), and expansion (72 °C) was implemented. Only for the assay targeting cg19283806 (*CCDC102B*), the annealing temperature was lowered to 51.5 °C for improved results.

**Table 1:** Correlations with Frailty Index in DNAm profiles of frail vs non-frail samples

	Unit	Correlation (Pearson r)	Correlation *p*-Value	Regression slope
Age	[years]	0.07 (− 0.247|0.373)	0.66939	0.002696
eFRS	–	0.291 (− 0.022|0.553)	0.06833	1.684188
cg19453093	[% DNAm]	− 0.384 (− 0.621|− 0.082)	**0.014441**	− 0.96458
cg13823169	[% DNAm]	− 0.08 (− 0.382|0.237)	0.621618	− 0.22673
cg20595453	[% DNAm]	− 0.411 (− 0.641|− 0.115)	**0.008359**	− 1.41193
cg19283806	[% DNAm]	− 0.257 (− 0.527|0.059)	0.109036	− 1.04174
cg22454769	[% DNAm]	0.119 (− 0.2|0.415)	0.463531	0.4499
cg17861230	[% DNAm]	− 0.04 (− 0.347|0.275)	0.806638	− 0.12452
ΔAge TaM clock	[years]	0.348 (0.041|0.595)	**0.02754**	0.004631
ΔAge TaC clock	[years]	0.273 (− 0.042|0.539)	0.088292	0.006933

In the DNAm profiles of 20 frail and 20 non-frail donors, we determined the Pearson’s correlation, correlation *p*-value, as well as the regression slope for a linear regression of the 32-item Frailty Index against chronological age, the eFRS, methylation at both targeted clocks’ sites, and the delta ages produced by their predictions. Statistically significant correlations are highlighted in bold

### Retraining TaM clock for dPCR data

Since DNAm levels measured via BeadChip analysis and dPCR showed systematic differences between these two methods, we retrained the TaM clock on dPCR measurements of 58 blood samples of 18 to 85 years (50% women), which included 32 samples of healthy younger donors that were collected and stored at the central biobank of the medical faculty of RWTH Aachen University (ethics approval number EK 206/09) [39] and 26 samples from the ActiFE study, which did not meet the FI-data inclusion requirement and therefore were only considered as part of this training set. The dPCR methylation measurements were used to train a new multivariate linear regression model for the TaM clock, using the *lm* function in R.

$$ \begin{aligned} {\text{TaM clock }}\left( {{\mathrm{dPCR}}} \right){ } & \approx { }152.32- 83.01 \times {\mathrm{cg}}19453093 \\ & \quad- 135.25 \times {\mathrm{cg}}13823169 \\ & \quad- 19.69 \times {\mathrm{cg}}20595453 \\ \end{aligned} $$

The TaC clock was adapted from our previous publications [17, 40] and derived from its original training data [14].

$$ \begin{aligned} {\text{TaC clock }}\left( {{\mathrm{dPCR}}} \right) & \approx 8.21 - 68.19 \times {\mathrm{cg}}19283806\\ &\quad + 91.29 \times {\mathrm{cg}}22454769 \\ & \quad+ 78.31 \times {\mathrm{cg}}17861230 \\ \end{aligned} $$

### Mortality risk and survival analysis

For this analysis, we directly adapted mortality records from 446 participants at the 12-year follow-up examination at the ActiFE Study. Mortality status and date of death were obtained from the local registration offices for all participants. Time-to-event for the Cox proportional hazards regression analysis was defined as the interval between the baseline evaluation and time-to-death or time-to-last-census as applicable. At the time of last census, 105 out of 446 participants (23.54%) had died. The function *coxph* from the *survival* package in R was used to perform the regression models for the delta ages of the TaM and TaC clocks and methylation at their constituent CpG sites, adjusted for age and sex. The adherence of covariates to the Cox-model’s assumption of proportionality over time was confirmed using the function *cox.zph* from the *survival* package for all models and graphic representations were plotted utilizing the *survminer* package in R [41].

### Association with Frailty-Index

The deviations of the predictions with the dPCR TaM and TaC clocks (ΔAge), as well as each of their corresponding CpGs (ΔDNAm, using the clocks’ training datasets to adjust for age), were investigated in relation to sex, the 32-item FI, and 23 out of the underlying 32 individual items (excluding binary items with no or single case in the cohort). For binary items, methylation or delta age were compared via two-tailed t-tests. For continuous items, correlation (Pearson’s r) and correlation p-value were calculated for their relationship to methylation or delta age using the *cor.test* function in R. Bonferroni-correction was used to correct for multiple testing of the individual items and sex.

## Results

### The mortality-associated three-CpG-signature may capture aspects of frailty

**Fig. 1:**
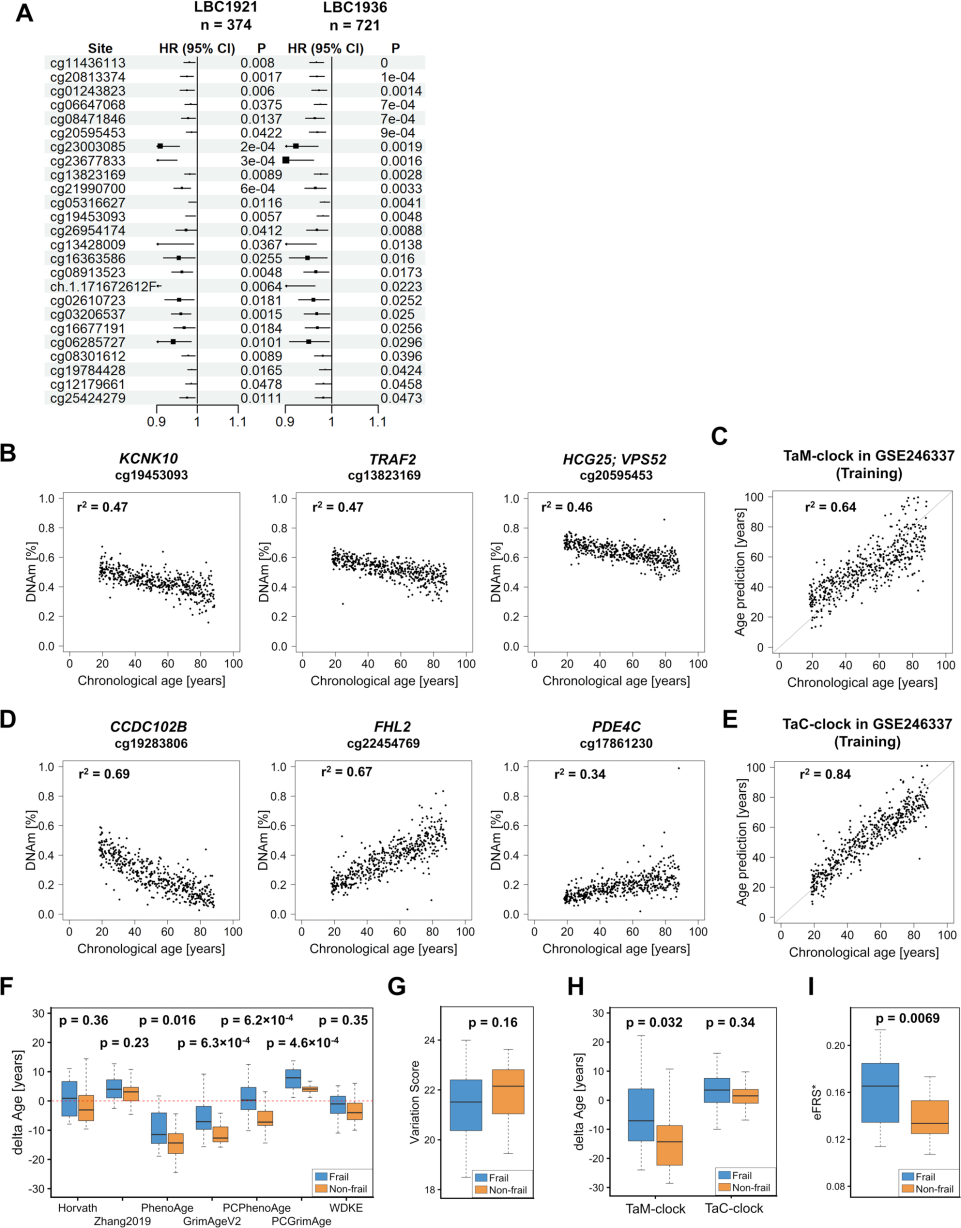
Derivation of the TaM and TaC clocks. **A** The selection of CpGs for a targeted mortality associated clock (TaM clock) was based on a subset of 25 CpGs that are associated with all-cause mortality in the Lothian Birth cohorts LBC1921 (*n* = 374 participants) and LBC1936 (*n* = 721) [16]. In all of these, hypomethylation is a significant risk factor associated with increased all-cause mortality (Cox-*p* < 0.05). **B** Of these 25 CpGs, we selected the three CpGs with highest age correlation (Pearson’s r^2^) in a training set of 500 healthy blood samples (GSE246337). **C** Multivariable linear regression was performed with these 3 CpGs to build the TaM clock (Pearson’s r^2^ = 0.64). **D** For comparison, we focused on three CpGs in the genes *CCDC102B*, *FHL2*, and *PDE4C*, that were previously identified for their very high correlation with chronological age [14, 17]. **E** Age correlation retraining a model utilizing these three age-associated CpGs (TaC clock) in the same training set (r^2^ = 0.84). **F** DNAm datasets of 20 frail and 20 non-frail samples of the ActiFE cohort were used for epigenetic age-predictions with the clocks of Horvath [32], Zhang et al., [33], PhenoAge [7],GrimAge V2 [8], PC-PhenoAge and PC-GrimAge [34], and the 27 CpG WDKE [16] (*p*-values were estimated by t-test). **G** As expected, the variation score of the WKDE-clock was in tendency higher for the non-frail samples. **H** Delta ages of the TaM and TaC clocks also revealed higher age-predictions in the frail samples compared to the non-frail ones. However, only the TaM clock revealed significant differences (*p* = 0.032). **I** The epigenetic frailty risk score [10] showed significantly higher scores in the frail subgroup (*p* = 0.0069)

The selection of mortality-associated CpGs was based on our previous analysis in the Lothian Birth Cohorts of 1921 and 1936 [16]. Notably, all the 25 CpG sites that clearly correlated with mortality in both cohorts (Fig. 1A) were hypomethylated with age, which is in line with our previous finding that particularly hypomethylated CpGs are associated with mortality [42]. To derive our mortality-associated TaM clock we focused on the three CpG sites with the highest correlation with chronological age in publicly available repository GSE246337. These were sites cg19453093 (annotated to the gene *KCNK10*), cg13823169 *(TRAF2),* and cg20595453 (*HCG25* and *VPS52*) (Fig. 1B). These three CpGs where then used to create a multivariable linear regression model for age predictions that provided a correlation with chronological age (Pearson’s r^2^) of 0.64 in the training set (Fig. 1C). For comparison, we employed our previously described targeted clock based on three CpGs that were only selected by correlation with chronological age (TaC clock) [17]: cg19283806 (*CCDC102B*), cg22454769 (*FHL2*), cg17861230 (*PDE4C*) (Fig. 1D), which provided a higher correlation with chronological age in the same training set (r^2^ = 0.84; Fig. 1E). Both the BeadChip-data-trained TaM and the TaC clocks were tested on a public dataset GSE40279, achieving a correlation with age (r^2^) of 0.39 (TaM) and 0.68 (TaC; Supplemental Fig. 1).

To evaluate how the blood cell composition might impact DNAm at the TaC and TaM sites, we analysed methylation profiles of isolated leucocyte populations. In fact, all sites showed quantitative differences between purified cell types, and this was particularly observed for cg19453093 and cg13823169 of the TaM clock (Supplemental Fig. 2). Thus, changes in the cellular composition may impact age-predictions with these clocks.

**Fig. 2:**
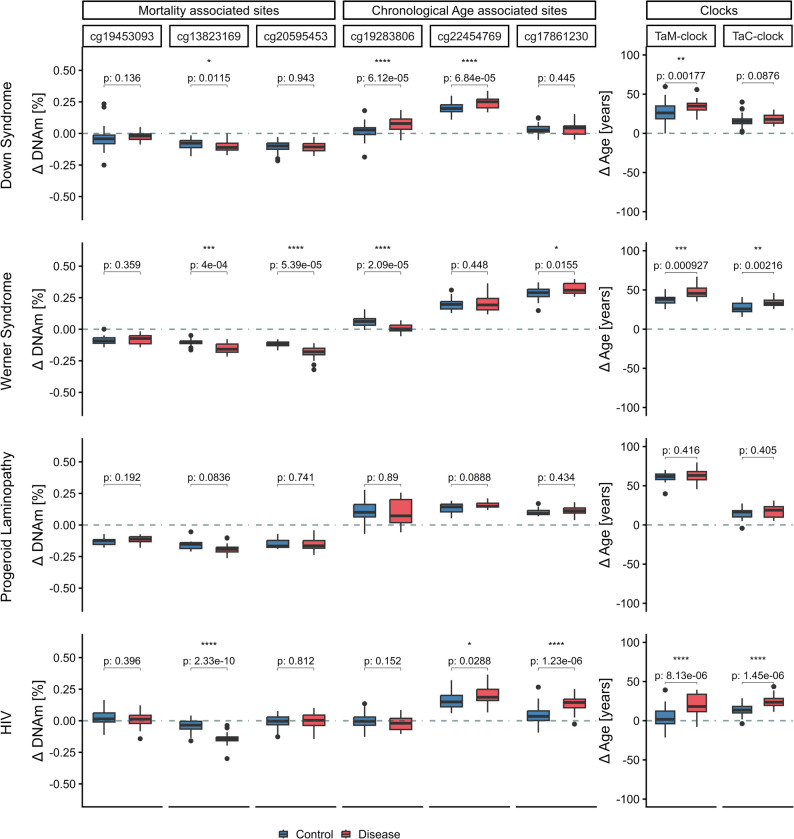
Targeted clock sites in diseases with accelerated aging. To determine how the CpGs of the TaM and TaC clocks are affected by diseases with accelerated aging, we used datasets of Down syndrome (GSE52588) [35], Werner Syndrome (GSE131752) [36], progeroid laminopathies (GSE182991) [37], and HIV (GSE67751) [38]. For individual CpGs of the TaM and the TaC clocks the deviation of DNAm levels as compared to age-adjusted values was calculated (ΔDNAm). Furthermore, for the three-CpG signatures the deviation of predicted and chronological age was determined (ΔAge). Means of diseased and corresponding control samples were compared via unpaired two-tailed Welch’s t-tests

For an initial evaluation of the relationship between epigenetic age-predictions and frailty, also in comparison with array-based epigenetic signatures, we analysed DNAm profiles of 20 frail and 20 non-frail participants of the ActiFE study with Infinium MethylationEPIC v2.0 BeadChips. We applied seven different epigenetic clocks to these samples (Fig. 1F). Despite the clear clinical differences between frail and non-frail participants, only the second-generation clocks PhenoAge (*p* = 0.016) and GrimAgeV2 (*p* = 6.3 × 10^–4^) as well as their PCA-counterparts (which calculate principal components from CpG-level data), PCGrimAge (*p* = 6.2 × 10^–4^) and PCPhenoAge (*p* = 4.6 × 10^–4^) revealed significant differences in the delta ages between frail and non-fail groups. In addition, the WKDE clock—a non-parametric epigenetic age predictor that does not require linear or logarithmic assumptions—provides a variability score that reflects the heterogeneity of age-associated changes. A lower variability score has been associated with shorter life-expectancy [16]. Conversely, the variability score was in tendency lower in the frail cohort (*p* = 0.16; Fig. 1G). A comparison of estimated blood cell compositions between the frail and non-frail cohort revealed no significant differences (Supplemental Fig. 3).

**Fig. 3:**
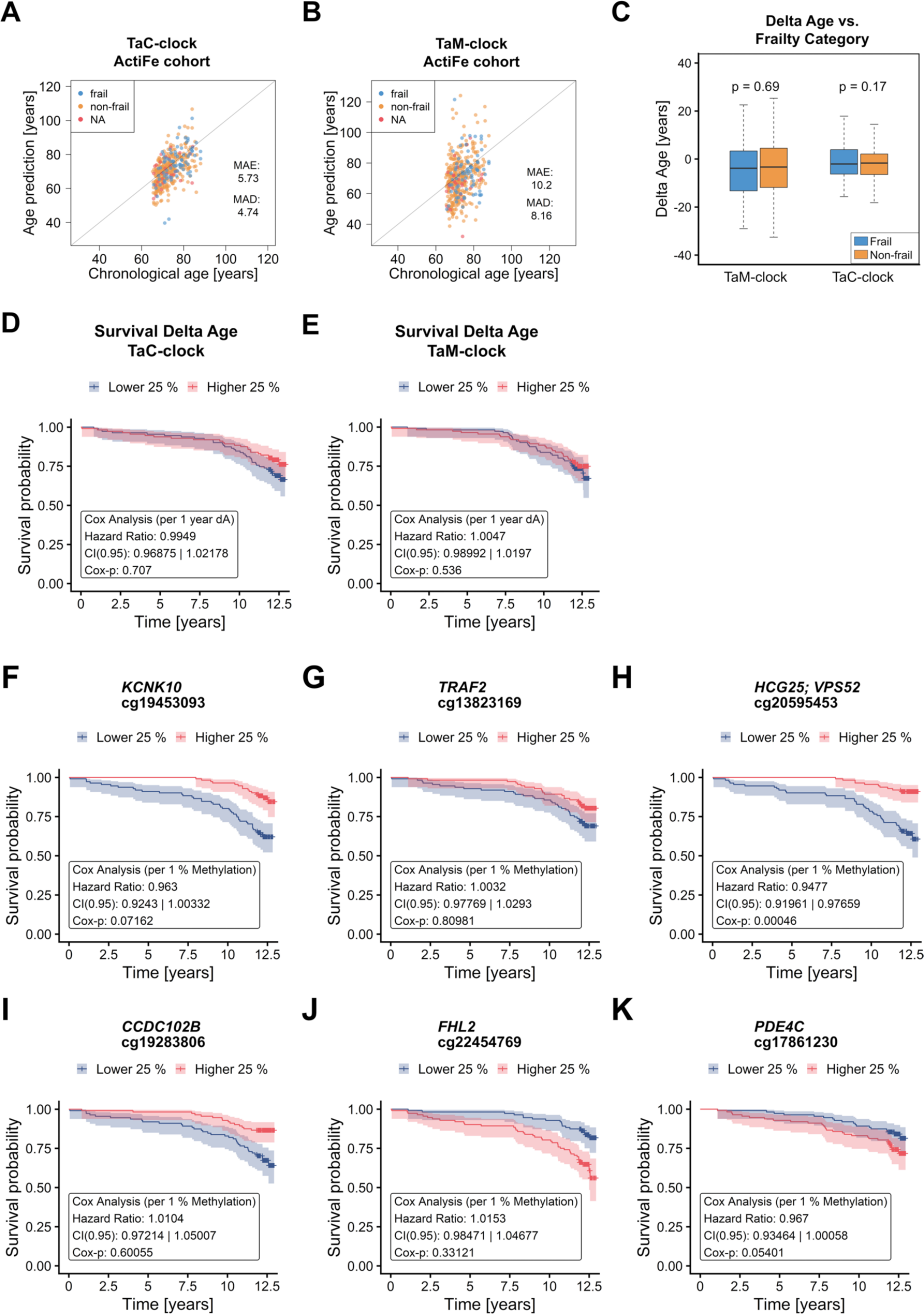
Targeted TaM and TaC clock predictions in the ActiFE cohort. **A**, **B** Age predictions by the dPCR based TaC and TaM clocks in 446 samples of the ActiFE cohort. **C** Delta ages in the frail *versus* non-frail donors calculated in both targeted clocks and compared via two-tailed t-test. **D**, **E** Kaplan–Meier (KM) plot and Cox regression analysis for delta age in the ActiFE cohort for the TaC clock (**D**) and the TaM clock (**E**) (KM-plots uncorrected for age and sex). **F–H)** KM-plot and Cox regression analysis for individual TaM CpG sites. **I–K** KM-plot and Cox regression analysis for individual TaC CpG sites (KM-plots uncorrected for age and sex)

Subsequently, we tested the BeadChip TaM and TaC clocks. Analogous to the much larger epigenetic signatures, they overall predicted the frail group to have accelerated age. However, only the TaM clock revealed a significant difference between the delta ages of frail and non-frail individuals (*p* = 0.032), while the TaC clock did not show this significant association of delta age and frailty category (*p* = 0.34; Fig. 1H). This supports the notion, that analogous to second-generation epigenetic clocks, our targeted signature based on mortality-associated CpGs can better capture aspects of frailty and biological condition than targeted signatures derived uniquely from associations with chronological age.

When we used the previously published epigenetic Frailty Risk Score (eFRS) [10] we observed that frail participants had significantly higher epigenetic frailty risk scores (*p* = 0.0069; Fig. 1I), with a moderate Pearson correlation of the eFRS with the 32-item FI of 0.291 (*p* = 0.068). Notably, for two CpGs of the TaM clock we observed a significant and slightly higher association with the 32-item FI for methylation: cg19453093 (r = − 0.384, *p* = 0.014) and cg20595453 (r = − 0.411, *p* = 0.0084), which was also observed for the TaM clock delta ages (r = 0.348, *p* = 0.028; Table 1). This further supports the idea, that the TaM clock may reflect some aspects of frailty and health.

### Evaluation of targeted epigenetic clocks on DNAm profiles of age-associated diseases

To further evaluate whether the TaM clock more accurately reflects aspects of aging than the TaC clock, we investigated datasets of Down syndrome [35], Werner syndrome [36], progeroid laminopathy [37], and HIV [38]. All of these diseases show clinical signs of accelerated aging [43–46]. Both of our three-CpG-clocks revealed accelerated epigenetic aging in patients with Werner-syndrome (TaM: *p* < 0.001, TaC: *p* = 0.002) and HIV (TaM: *p* < 10^–5^, TaC: *p* < 10^–5^; Fig. 2). In patients with Down syndrome only the TaM clock revealed significant age-acceleration (*p* < 0.002), while in progeroid laminopathy neither of the two clocks reached significance. In addition, we also analysed the deviation of age-related DNAm levels (ΔDNAm), at the individual CpGs of the TaM and TaC clocks of which several showed significant differences in disease *versus* controls, although without consistent pattern. Overall, the TaM clock appeared to be more sensitive to detect age-acceleration in these diseases, and we observed that individual CpGs in both signatures indicated accelerated age for specific conditions.

### Analysis of targeted epigenetic clocks via digital PCR in the ActiFE cohort

Subsequently, we established dPCR assays to allow cost-effect and reliable measurements of DNAm levels. When we compared the DNAm levels of dPCR with the BeadChip results in the above mentioned 20 frail and 20 non-frail samples, we observed high correlation of the results, but all three assays revealed notable and systematic deviations in absolute measured values (Supplemental Fig. 4A). We therefore retrained the multivariable model for the TaM clock for dPCR measurements (Supplemental Fig. 4B, C). For the TaC clock, we utilized the previously established dPCR model, which was also used on other ongoing studies (Supplemental Fig. 4D).

In total, we analysed 446 samples from the ActiFe cohort via dPCR, measuring the 6 CpGs of both targeted clocks. As anticipated, predictions made with the TaC clock were more accurate with a mean absolute error (MAE) of 5.73 years and a median absolute deviation (MAD) of 4.74 years (Fig. 3A), whereas the deviations were higher for the TaM clock (MAE = 10.2 years, MAD = 8.16 years; Fig. 3B). While DNAm at two TaM sites (cg19453093: *p* = 0.024; cg20595453: *p* = 5.28 × 10^–5^) and TaC sites (cg19283806: *p* = 0.0069; cg22454769: *p* = 1.4 × 10^–4^) was significantly associated with the 32-item FI, delta ages of neither targeted clock showed such correlation (TaM: *p* = 0.905; TaC: *p* = 0.186874; Table 2). Similarly, when we stratified the analysis by frailty status, both targeted clocks did not reveal significant differences in mean delta ages between the two groups (Fig. 3C). Furthermore, we investigated the correlation of individual items of the FI against the targeted clocks or the age-corrected DNAm at their sites. After adjusting p-values for multiple testing, only the item ‘daily number of medicines taken’ showed significant association with DNAm at cg20595453 (Supplemental Tables 2 and 3). For the overall 32-item FI only cg20595453 (*p* = 0.0023) and cg22454769 (*p* = 0.0421) showed statistically significant correlation after correcting for age.

**Table 2:** Correlations between Frailty Index and DNAm profiles in the population-based cohort

	Unit	Correlation (Pearson r)	Correlation* p*-Value	Regression slope
Age	[years]	0.258 (0.166|0.345)	**9.25 × 10**^**–8**^	0.004586
cg19453093	[% DNAm]	− 0.111 (− 0.204|− 0.015)	**0.02384**	− 0.23035
cg13823169	[% DNAm]	− 0.063 (− 0.158|0.034)	0.201427	− 0.07729
cg20595453	[% DNAm]	− 0.196 (− 0.287|− 0.102)	**5.28 × 10**^**–5**^	− 0.31584
cg19283806	[% DNAm]	− 0.132 (− 0.225|− 0.037)	**0.006904**	− 0.22334
cg22454769	[% DNAm]	0.185 (0.091|0.276)	**0.000139**	0.257591
cg17861230	[% DNAm]	0.042 (− 0.054|0.137)	0.391418	0.076352
ΔAge TaM clock	[years]	− 0.006 (− 0.102|0.09)	0.904534	− 4.33 × 10^–5^
ΔAge TaC clock	[years]	0.065 (− 0.031|0.16)	0.186874	0.000859

In the 446 samples of the ActiFE cohort, we determined the Pearson’s correlation, correlation *p*-value, as well as the regression slope for a linear regression of the 32-item Frailty Index against chronological age, methylation at both dPCR based clocks’ sites, and the delta ages of epigenetic age predictions. Statistically significant correlations are highlighted in bold

Next, we analysed the association of epigenetic age predictions with all-cause mortality in the ActiFE cohort via Cox regression, corrected for age and sex. There was no clear association of delta age with neither the TaC clock (HR = 0.995 per year delta age, *p* = 0.707; Fig. 3D), nor with the TaM clock (HR = 1.005 per year delta age, *p* = 0.536; Fig. 3E). This was somewhat unexpected, since the TaM clock was specifically trained on CpGs associated with mortality. We therefore further evaluated how the individual TaM and TaC sites were associated with all-cause mortality, again using adjusted Cox proportional hazards regression models (Fig. 3F–K). In fact, particularly cg20595453 of the TaM clock was highly significantly associated with all-cause mortality (HR = 0.948, 95%CI = (0.920|0.977), Cox-*p* = 4.6 × 10^–4^). Additionally, a trend towards association with mortality was observed for cg19453093 of the TaM clock (HR = 0.963, 95%CI = (0.9243|1.003), Cox-*p* = 0.072) and cg17861230 of the TaC clock (HR = 0.967, 95%CI = (0.935|1.001), Cox-*p* = 0.054). However, the multivariable model of the TaM clock—weighted for accurate chronological age prediction—more heavily factored methylation at site cg13823169, which showed poor association with mortality in the ActiFE cohort (HR = 1.0032, 95%CI = (0.9777|1.0293), Cox-*p* = 0.810).

## Discussion

Within the last 15 years a multitude of epigenetic signatures have evolved for a wide spectrum of aging concepts, including chronological age, frailty, biological age and mortality. So far, however, these measures have not seen widespread clinical adoption and it remains to be proven if such measures can be of diagnostic or therapeutic relevance. While it is evident that information on the individual aging process can be gathered from observing DNAm changes, practical hurdles remain that hinder the translation of epigenetic signatures into clinical tools.

Intuitively, larger signatures of hundreds of CpGs appear to be more robust and reliable, since they may better capture the bandwidth of age-associated physiological changes and their potential impact on the epigenetic landscape. However, analysis of genome wide DNAm profiles is difficult to implement into clinical settings, due to costs, time, and legal constrains. Furthermore, the commonly used Illumina BeadChip platforms are not certified for clinical application in Europe and certification of complex analysis algorithms presents significant challenges for approval as in vitro diagnostics device (IVDD) [12]. Therefore, targeted signatures based on few individual CpGs, which can be measured e.g. by digital PCR or pyrosequencing, appear attractive.

Previously, other studies have also identified individual mortality or frailty associated CpGs, however, to our knowledge these have not been further developed into targeted signatures to be measured independent of the Illumina BeadChip platform [10, 42, 47–50]. In this regard, our study went a step further in that we established measurement by digital PCR, which may facilitate more reliable DNAm measurements since there is no PCR bias between methylated and non-methylated sequences [14, 39]. Furthermore, such measurement is feasible within 6 h and can be applied for dried blood spots, which can easily be harvested by a finger prick [39, 51]. Thus, the TaM and TaC clocks could be easily implemented into clinical practice—if they provide relevant information for personalized medicine.

The established second-generation clocks, such as GrimAge and PhenoAge, are conceptually not predictors of chronological age. They are trained directly to reflect relevant biological endpoints, modelling methylation-based predictions of clinical parameters such as pack-years, cystatin C, or leptin in GrimAge V2 [8], or albumin, creatinine, and erythrocyte size distribution in PhenoAge [7]. As such, they may be capturing DNAm changes, which are indicative of morbidity rather than capturing aging trajectories. Thus, it might be misleading to consider these signatures as true age predictors. Our TaM clock also aims to capture aspects beyond chronological age, but since we did not include CpGs for their association with specific age-associated parameters, it would be overstated to consider this three CpG signature as a second-generation clock.

Overall, our results indicate that the TaM clock could indeed improve upon the TaC clock in capturing aspects of mortality, morbidity, and frailty. While both the TaM and TaC clocks did not reflect epigenetic age-acceleration in progeroid laminopathy this was also not observed with other epigenetic clocks before, such as the Horvath pan-tissue clock and the Horvath skin and blood clock, when they were applied to blood samples [37, 52]. Yet, in skin fibroblasts of donors, the Horvath skin and blood clock might reveal some age-acceleration in younger patients with Hutchinson-Gilford progeria [53]. Taken together, incorporating CpG sites associated with both age and mortality may render the TaM clock more reflective of biological aging than clocks based solely on age-associated sites. However, similar to second-generation epigenetic clocks, this comes with a trade-off regarding prediction accuracy to individual chronological age [54, 55].

The dPCR models for the TaM and TaC clocks were trained on relatively small cohorts, and it is conceivable that their performance could be further improved with larger training datasets. However, we have previously demonstrated that targeted epigenetic age predictors based on pyrosequencing or dPCR can achieve remarkable accuracy in independent validation cohorts despite being trained on as few as 84, 24, or even 12 samples [13, 56, 57]. Biological age, however, is likely a more complex construct than chronological age [7, 58]. Therefore, targeted predictors of biological age may require larger training cohorts and/or a greater number of methylation sites than chronological age predictors to adequately capture complex phenotypes such as frailty and mortality risk. Future studies should systematically investigate how training sample size and marker selection influence the performance and robustness of targeted biological age predictors.

Particularly the TaM clock site cg20595453 seemed to be significantly associated with mortality, frailty, and the daily number of medicines taken (as a surrogate for disease burden of the 32-item FI). This CpG is annotated to the lncRNA *HCG25* and the gene *VPS52*, which is part of the GARP-complex in trans-Golgi endosomal transport [59]. *HCG25* and *VPS52* do not seem to show age-associated differential gene expression [60]. Another TaM clock CpG, cg19453093, is linked to a putative enhancer within KCNK10 [61], which encodes the voltage- and mechanosensitive potassium channel TREK-2, and this gene showed moderate age-associated down-regulation of gene expression in a previous study [60]. Furthermore, cg13823169 is associated with a putative enhancer of *TRAF2*, which encodes TNF receptor-associated factor 2, an E3 ubiquitin ligase that plays a central role in TNF-α-mediated signaling pathways regulating cell survival and apoptosis [62]. While these observations may suggest that differential DNAm at the TaM sites has biological relevance, the functional consequences of these methylation changes remain unclear, and the signature should primarily be regarded as a biomarker rather than a mechanistically validated driver of biological aging. This does not preclude that in general, the age-associated changes in the epigenetic landscape are relevant for the aging process and that the DNAm at these clock sites may be involved in a coordinated epigenetic network [63, 64].

There is growing evidence that epigenetic clock predictions are influenced, at least in part, by age-related changes in cellular composition [65, 66]. Consistent with this notion, we observed DNAm differences at both the TaM and TaC sites. Although age-associated DNAm changes were also detectable in purified cell subsets, it remains plausible that shifts in cellular composition contribute substantially to changes in epigenetic age estimates. For small targeted epigenetic clocks, adjustment for cell-type composition is particularly challenging unless additional CpG sites are incorporated that specifically capture cellular heterogeneity [39, 67]. From the perspective of a biomarker of biological age, however, it may be less critical whether age predictions primarily reflect intrinsic molecular aging processes or age-associated alterations in cellular composition. Similar to second-generation epigenetic clocks, the inclusion of CpGs that capture clinically relevant parameters, such as leukocyte composition, may even enhance the biological and clinical utility of these predictors.

Notably, the TaM clock captured differences between the DNAm profiles of individuals with the highest and lowest frailty scores in the ActiFE cohort, but failed to discriminate frailty state in the wider cohort of 446 samples when analysed by dPCR. We hypothesize that the lessened effect observed in the larger cohort resulted from the preselection criteria applied to the initial 40 samples, which were intentionally chosen based on greater differences in the frailty indices. It needs to be taken into account that the ActiFE cohort consists only of non-institutionalized older adults, making this cohort likely comparatively healthy and potentially reducing variability in frailty phenotype [23]. In the preselected 40 DNAm profiles, our TaM clock showed similar association with frailty-index scores as the previously described epigenetic Frailty Risk Score (eFRS) [10]. Furthermore, even this specifically trained signature for frailty revealed only moderate correlation between eFRS and the frailty index at baseline and various follow-ups [10]. Given that such correlation coefficients of eFRS, TaM clock, and TaC clock were modest (maximum varying around 0.3), none of the models seems well-positioned to substitute a clinical frailty assessment based on the frailty index. An epigenome wide association study (EWAS) conducted in the LBC1936 found only one site of marginal significance for frailty [68] and even many of the larger second generation clocks do not reliably capture frailty [69]. Thus, there may be general limitations in how the complex clinical and physiological parameters, which contribute to the concept of frailty, are reflected by epigenetic changes in blood.

## Conclusions

Targeted epigenetic signatures hold great potential for clinical applications. Our TaM clock revealed better association with frailty than the TaC clock in the 40 Illumina BeadChip profiles of frail and non-frail donors, indicating that it is in principle possible to further optimize such signatures. However, particularly when investigating a relatively healthy population, our model’s predictive value for frailty and mortality is low. It is conceivable that the rate of change in these clocks over time provides additional insight into aging trajectories, as recently suggested in the InCHIANTI cohort [70]. Furthermore, the predictive power might be improved by integrating additional CpG sites that are associated with specific diseases or clinical parameters. Measurement by dPCR is feasible within few hours and can even be applied to dried blood spots, harvested by a finger prick. To this end, our study contributes to the growing body of developments aiming to bring epigenetic analysis into clinical application.

## Supplementary Information

Below is the link to the electronic supplementary material.

Supplementary Material 1

## Data Availability

DNA methylation datasets are available upon reasonable request and upon approval by the ActiFE study group.
