# Supplementary material for: Exploring a targeted epigenetic clock based on mortality-associated CpGs as a potential biomarker for frailty
Source: Clin Epigenetics. 2026 Jul 7;18:132. doi: 10.1186/s13148-026-02196-9 (PMC13339440; doi:10.1186/s13148-026-02196-9)
Supplement: Supplementary file 1 — Supplementary Material 1 [file 13148_2026_2196_MOESM1_ESM.pdf]

Supplementary Information

Exploring a targeted epigenetic clock based on mortality-associated CpGs as a potential biomarker for frailty

Jonathan Awuah, Dhayana Dallmeier, Felix Boehm, Laura Hertle, Dietrich Rothenbacher, Juan-Felipe Perez-Correa, Wolfgang Wagner

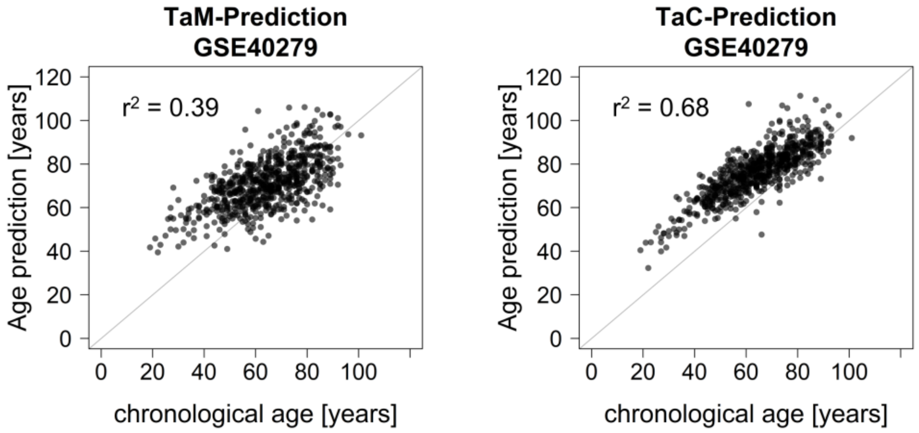

Supplemental Figure 1: Testing of the BeadChip clocks on an independent dataset.

Application of the BeadChip TaM and TaC clocks in an independent dataset containing n=656 samples of donors aged 19 to 101 years. As anticipated, the correlation values are lower than in the training cohort. Furthermore, the TaC clock achieved a higher correlation with chronological age than the TaM clock.

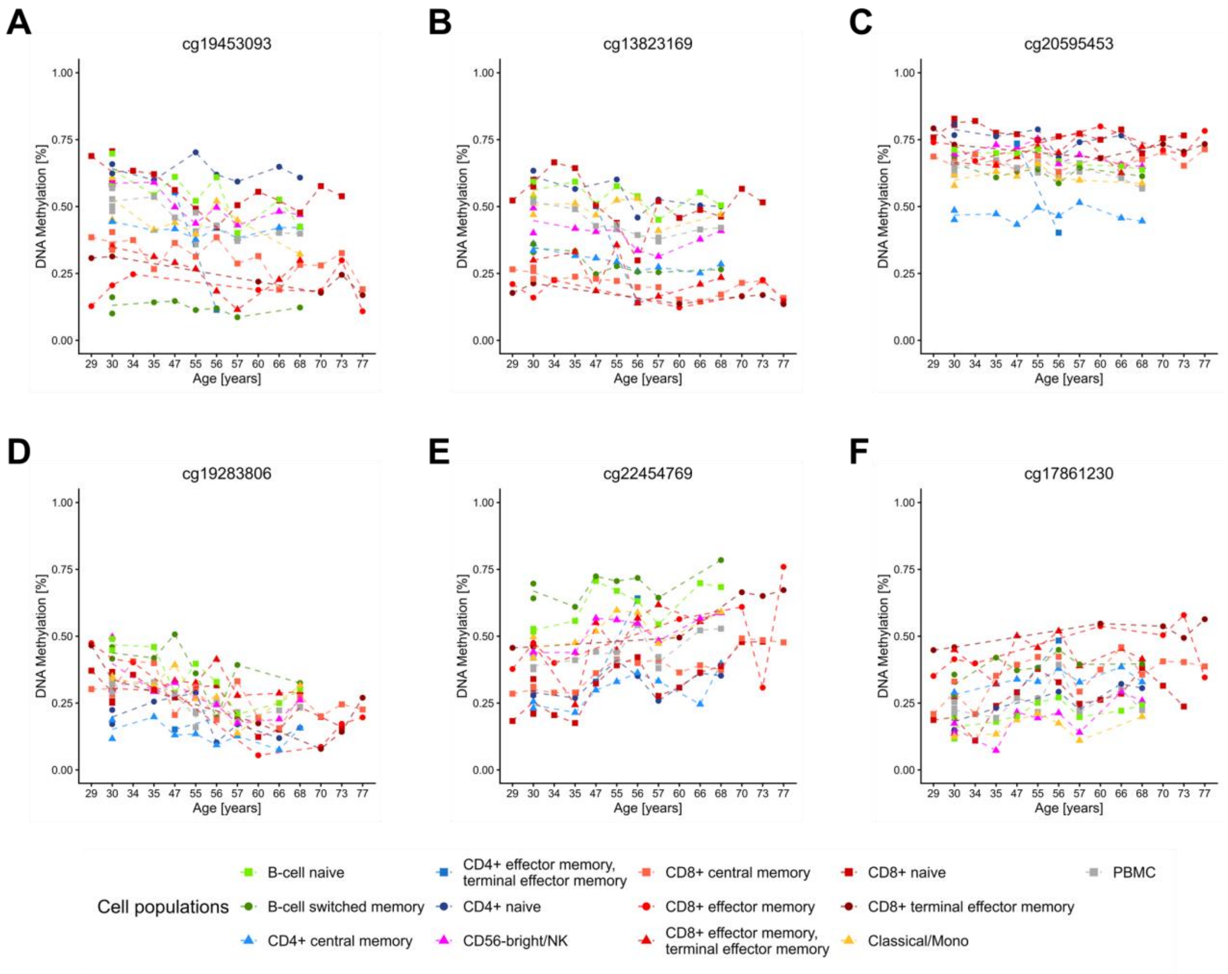

Supplemental Figure 2: Investigating TaM and TaC sites in different leucocyte subsets.

The three CpGs of **A-C)** the TaM clock, and **D-F)** the TaC clock were analyzed in a dataset of purified leucoytes subpopulations (GSE252045). This dataset revealed for all CpGs clear differences in DNAm levels between cell types. The age-associated changes showed overall similar slopes for individual leucocyte subsets, whereas cg20595453 showed relatively little age-association in this small cohort.

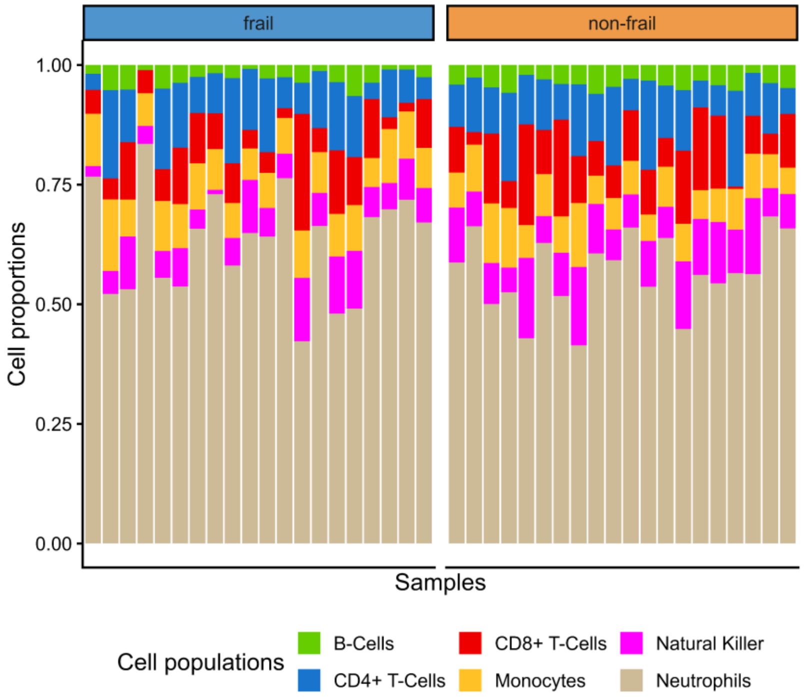

Supplemental Figure 3: Cell composition estimates in frails *versus* non-frail samples.

Estimated blood cell proportions for the 40 samples measured via BeadChip array using the ENmix implementation of methylation-based cell type deconvolution by Houseman et al. (2012). In tendency, frail samples showed slightly reduced B- and NK-cells and increased neutrophile fractions, but these differences did not reach statistical significance when adjusting for multiple comparisons.

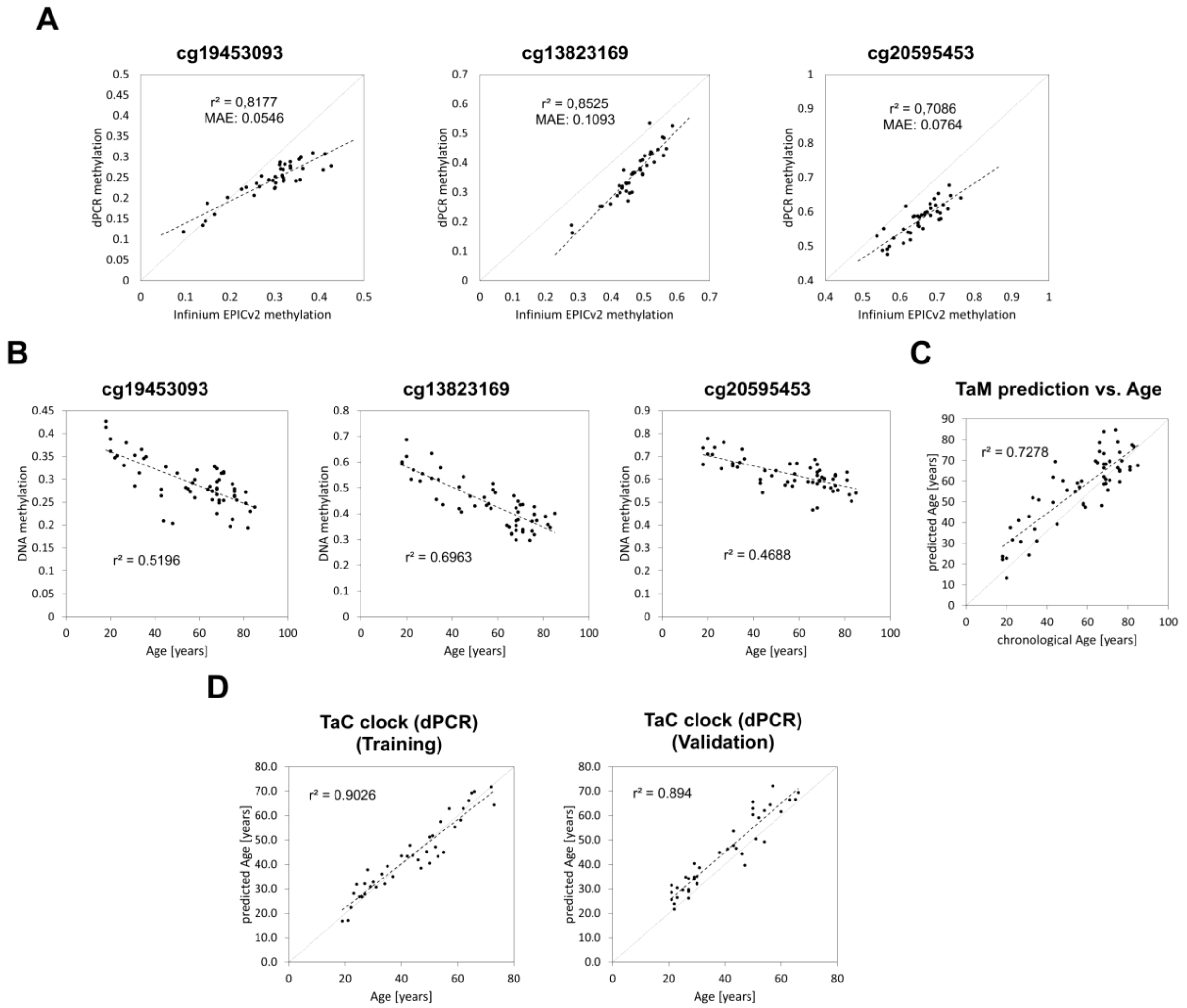

Supplemental Figure 4: Retraining of TaM and TaC clocks for dPCR measurements.

A) DNA methylation levels at the CpGs of the TaM clock were compared by in 40 samples of the ActiFE cohort by Infinium EPICv2 array and digital PCR measurement. While the correlation was overall very high, there was a clear offset. Pearson´s correlation (R^2) and median absolute error (MAE) are indicated. **B)** Correlation of the dPCR measurements with chronological age in 58 blood samples (18 to 85 years) revealed high correlation for all CpGs. These measurements were used to train a new multivariate linear regression model for the TaM clock. **C)** Performance of the TaM dPCR model in the training set. **D)** For the TaC clock, we used a dPCR model that was trained and validated on independent datasets, as previously described in Han et al. (*BMC Biol*; 2020: https://doi.org/10.1186/s12915-020-00807-2).

Supplemental Table 1: Primers and probe design for digital PCR

Site/ probe designation EPICv2	Sequence
**cg19453093_TC21 (reverse strand)**	
Forward Primer	5’-TGTAGGATTGATTGTTGA-3’
Reverse Primer	5’-CACCCTTCTAAATATCCC-3’
Probe Methylated	6-Fam -TTTATAATATTTCGTAATTTTGTTTGGGA-MBQ-1
Probe Unmethylated	Hex -TTTATAATATTTTGTAATTTTGTTTGGGAG-MBQ-1
**cg13823169_TC21 (forward strand)**	
Forward Primer	5’-TTAGTGAGTGGTTGGTA-3’
Reverse Primer	5’-CACATACAACTACTATCCTA-3’
Probe Methylated	6-Fam -TGGTTTATGGTTATGTCGTT-MBQ-1
Probe Unmethylated	Hex -TGTGGTTTATGGTTATGTTGTT-MBQ-1
**cg20595453_TC21 (Forward strand) Genes: *HCG25; VPS52***	
Forward Primer	5’-TTTGAAGTTGGTGAAATTT-3’
Reverse Primer	5’-ACACTATTTCCTCCTACT-3’
Probe Methylated	6-Fam -ATTATTAAAGTTACGGATTAGTTGAGTT-MBQ-1
Probe Unmethylated	Hex -AATTATTAAAGTTATGGATTAGTTGAGTTA-MBQ-1
**cg22454769_TC11 (reverse strand) Gene: *FHL2***	
Forward Primer	5’-GTGGGTAGATTTTTGTTAT-3’
Reverse Primer	5’-CTTTATTTACCAAAACTCCT-3’
Probe Methylated	6-Fam -TTTTCGATAATTATTGTGTTTTTAAGATT-MBQ-1
Probe Unmethylated	Hex -TTTTTGATAATTATTGTGTTTTTAAGATT-MBQ-1

Supplemental Table 2: Association of the TaM-clock CpGs with parameters of the frailty index

	ΔDNAm cg19453093		ΔDNAm cg13823169		ΔDNAm cg20595453		ΔAge TaM	
**Binary Values**	Mean-diff.	t-Test p	Mean-diff.	t-Test p	Mean-diff.	t-Test p	Mean-diff.	t-Test p
Sex (M vs. F)	-0.0108	0.1968	-0.0168	0.3986	-0.0089	1	3.1453	0.2352
Alone	0.0024	1	0.0015	1	-0.0036	1	-0.8191	1
Fall last 12 months	-0.0026	1	-0.0029	1	-0.0046	1	0.4321	1
Hypertension	-0.0029	1	-0.007	1	-0.012	0.5637	1.2933	1
Myocardial Infarction	0.001	1	0.0058	1	-0.0123	1	-0.9409	1
Heart Failure	0.0017	1	0.0169	1	-0.013	1	-2.4952	1
Stroke	0.0114	1	0.0244	1	-0.0141	1	-4.9532	1
Cancer	-0.004	1	0.0163	1	-0.0072	1	-2.2186	1
Diabetes	-0.0022	1	-0.016	1	-0.0123	1	2.432	1
Rheumatism	0.0059	1	-0.0026	1	-0.0072	1	-0.0643	1
COPD	-0.0025	1	0.0195	1	3.00E-04	1	-1.4445	1
Migraines	0.0015	1	0.0081	1	0.0058	1	-0.7934	1
Incontinence	-0.0027	1	0.0044	1	-0.0037	1	-0.4509	1
**Continuous Values**	r	Coef. p	r	Coef. p	r	Coef. p	r	Coef. p
Systolic blood pressure	-0.0329	1	-0.0478	1	-0.0876	1	0.0513	1
Diastolic blood pressure	-5.0×10^{-4}	1	0.0177	1	-0.0628	1	0.0166	1
BMI	0.0349	1	-0.0451	1	-0.0219	1	0.03	1
Medicine intake per day	-0.0448	1	-0.0028	1	-0.1585	0.0187	-3.0×10^{-4}	1
HADS Anxiety Score	0.0212	1	0.0117	1	0.046	1	-0.0186	1
HADS Depression Score	-0.0035	1	-0.0317	1	-0.0146	1	0.0133	1
Lubben Social Network Scale score	0.1041	0.6863	0.0586	1	0.0391	1	-0.0695	1
Mini Mental State Examination	0.1147	0.4661	0.0649	1	0.0591	1	-0.0767	1
Grip Strength	-0.0661	1	-0.0701	1	-0.0026	1	0.0967	1
Gait Speed	-9.0×10^{-4}	1	0.0165	1	0.0725	1	0.0076	1
Forced chair rise time	0.0042	1	0.0636	1	-0.0419	1	-0.0652	1
**32 item Frailty Index (p unadjusted)**	-0.0565	0.249	0.0074	0.8794	-0.1486	0.0023	-0.0059	0.9045

Supplemental Table 3: Association of the TaC-clock CpGs with parameters of the frailty index

	ΔDNAm cg19283806		ΔDNAm cg22454769		ΔDNAm cg17861230		ΔAge TaC	
**Binary Values**	Mean-diff.	t-Test p	Mean-diff.	t-Test p	Mean-diff.	t-Test p	Mean-diff.	t-Test p
Sex (M vs. F)	1.0×10^{-4}	1	0.0026	1	0.0176	0.0048	1.5123	0.624
Alone	-0.0036	1	0.0031	1	-0.0053	1	-0.0394	1
Fall last 12 months	-0.0043	1	0.003	1	-1.0×10^{-4}	1	0.4041	1
Hypertension	0.0052	1	0.003	1	0.0058	1	0.3708	1
Myocardial Infarction	0.0248	1	0.0197	1	0.0048	1	0.387	1
Heart Failure	-0.0073	1	-9.0×10^{-4}	1	-0.0176	0.0888	-1.0633	1
Stroke	-0.0014	1	-0.0029	1	0.008	1	0.1242	1
Cancer	-0.0044	1	-0.0061	1	-0.0068	1	-1.1028	1
Diabetes	-0.0077	1	-0.0049	1	-0.0024	1	-0.1522	1
Rheumatism	-0.0026	1	-0.0127	1	-0.0022	1	-1.1552	1
COPD	0.0102	1	1.0×10^{-4}	1	0.0547	1	3.9708	1
Migraines	-0.0055	1	0.0047	1	-0.008	1	0.3979	1
Incontinence	-0.0052	1	-0.005	1	-0.0088	1	-0.9063	1
**Continuous Values**	r	Coef. p	r	Coef. p	r	Coef. p	r	Coef. p
Systolic blood pressure	0.0128	1	0.0689	1	-0.0423	1	0.02168	1
Diastolic blood pressure	-0.0135	1	-0.044	1	-0.0745	1	-0.05106	1
BMI	0.0419	1	0.0786	1	0.0713	1	0.08332	1
Medicine intake per day	0.0022	1	0.0583	1	-0.0173	1	0.01828	1
HADS Anxiety Score	0.0188	1	0.0357	1	0.0242	1	0.03123	1
HADS Depression Score	0.0081	1	0.1072	0.5955	0.061	1	0.1051	0.6719
Lubben Social Network Scale score	0.0137	1	-0.0808	1	-0.0095	1	-0.07213	1
Mini Mental State Examination	0.0361	1	-0.1147	0.4652	-0.0685	1	-0.1451	0.0741
Grip Strength	-0.0139	1	-0.0442	1	0.1275	0.1784	0.05644	1
Gait Speed	-0.0498	1	-0.0799	1	0.0107	1	-0.01604	1
Forced chair rise time	0.0213	1	0.1235	0.2468	0.0211	1	0.08894	1
**32 item Frailty Index (p unadjusted)**	-0.0573	0.2433	0.0994	0.0421	-0.0474	0.3334	0.06476	0.1869
